# Adaptations of white spruce to climate: strong intraspecific differences in cold hardiness linked to survival

**DOI:** 10.1002/ece3.3796

**Published:** 2018-01-08

**Authors:** Jaime Sebastian‐Azcona, Uwe G Hacke, Andreas Hamann

**Affiliations:** ^1^ Department of Renewable Resources University of Alberta Edmonton AB Canada

**Keywords:** assisted migration, cold hardiness, drought, plant–climate interactions, seed transfer, white spruce, wood anatomy, xylem

## Abstract

Understanding local adaptation of tree populations to climate allows the development of assisted migration guidelines as a tool for forest managers to address climate change. Here, we study the relationship among climate, a wide range of physiological traits, and field performance of selected white spruce provenances originating from throughout the species range. Tree height, survival, cold hardiness, hydraulic, and wood anatomical traits were measured in a 32‐year‐old common garden trial, located in the center of the species range. Provenance performance included all combinations of high versus low survival and growth, with the most prevalent population differentiation for adaptive traits observed in cold hardiness. Cold hardiness showed a strong association with survival and was associated with cold winter temperatures at the site of seed origin. Tree height was mostly explained by the length of the growing season at the origin of the seed source. Although population differentiation was generally weak in wood anatomical and hydraulic traits, within‐population variation was substantial in some traits, and a boundary analysis revealed that efficient water transport was associated with vulnerable xylem and low wood density, indicating that an optimal combination of high water transport efficiency and high cavitation resistance is not possible. Our results suggest that assisted migration prescriptions may be advantageous under warming climate, but pronounced trade‐offs between survival and cold hardiness require a careful consideration of the distances of these transfers.

## 1 INTRODUCTION

Geographic patterns of local adaptation of forest trees have been studied since the 18th century, and most studies found that local populations were the best fit to a specific environment (Langlet, 1971). This has led forest managers to develop so‐called seed zones, where areas of similar conditions were defined, assuming that individuals coming from that area would have superior growth and survival when planted within the same seed zone. More recently, climate change has led to locally adapted populations lagging behind their optimal climate niche, thus challenging the assumption that “local is best” (Aitken, Yeaman, Holliday, Wang, & Curtis‐McLane, 2008; Davis & Shaw, 2001). Gray and Hamann (2013) found that based on observed climate trends, forest tree species in western North America already lag behind their optimal climate niche by approximately 130 km in latitude. Furthermore, Alberta also experienced a reduction in precipitation in the past 25 years (Mbogga, Hamann, & Wang, 2009), and the trend toward drier conditions may continue during the 21st century (Wang, Hogg, Price, Edwards, & Williamson, 2014). As a consequence, trees may become increasingly maladapted to new climate conditions.

One way of accommodating changes in climate is the use of seed sources from areas already adapted to warmer temperatures as part of regular reforestation programs. This usually implies selecting seeds from southern areas to be planted in a more northern region (Millar, Stephenson, & Stephens, 2007). Such assisted migration prescriptions depend on identifying well‐adapted genotypes from matching climate regions. This can be carried out using provenance trials, in which seed sources collected from different geographic regions and different environments are planted in a common garden where genetic differences between populations may be observed. If promising genotypes can be identified in provenance trials, then these genotypes could be moved and planted where their characteristics match the anticipated climate. Previous work on white spruce provenances in different parts of its distribution suggests that transfers toward the north can increase growth rates (e.g., Gray et al., 2016; Lesser & Parker, 2004; Li, Beaulieu, & Bousquet, 1997; Lu et al., 2014; Rweyongeza, Yang, Dhir, Barnhardt, & Hansen, 2007). A recent study proposed relatively short northward transfers for Alberta, with growth and survival of transferred seed sources putatively limited by cold temperatures in the north of the province (Gray et al., 2016). All these studies analyzed the response of tree growth to different planting environments, but there is little understanding of the physiological causes of different local adaptations.

In the boreal forest, increased temperatures may have a positive effect on tree growth, as has been observed in some white spruce populations (Danby & Hik, 2007; Lloyd & Fastie, 2002; MacDonald, Szeicz, Claricoates, & Dale, 1998). But this positive effect will only occur with adequate water availability, as the opposite effect was found in drier areas or years, showing that drought can be an important limitation for white spruce development in the future (Barber, Juday, & Finney, 2000; Chen et al., 2017; Danby & Hik, 2007; Jiang, Huang, Stadt, Comeau, & Chen, 2016; Lloyd & Fastie, 2002). Even if precipitation rates are not affected by climate change, increased temperatures will enhance drought stress in plants by increasing transpiration. With higher transpiration, water reserves will deplete faster resulting in a heat‐induced drought (Breshears et al., 2005). Moreover, snow reserves will melt earlier, further reducing water availability later in the growing season (Barnett, Adam, & Lettenmaier, 2005). Finding a productive and drought‐resistant genotype might be a difficult challenge as trade‐offs between growth and heat/drought resistance have been reported in white spruce (Bigras, 2000, 2005). The trade‐off between hydraulic safety and efficiency of the xylem was analyzed in detail by Gleason et al. (2016), arriving at the conclusion that although the correlation between both traits is not always clear, the combination of both high efficiency and high resistance is not possible. This trade‐off can be partially explained by anatomical features of the tree such as wood density, conduit size, or ratio between photosynthetic and conductive tissue (Gleason et al., 2016; Sperry, Hacke, & Pittermann, 2006).

Even though frost events are lower in frequency and severity under recent climate warming, extreme cold events may still occur on rare occasions, especially if overall variability in climate increases. A single unexpected frost event can cause great damage to forests if it occurs after the start of the growing season (Gu et al., 2008). Man, Kayahara, Dang, and Rice (2009) also reported severe frost damage in a white spruce stand after a late spring frost. As such frost events that cause dieback and mortality are rare, it remains difficult to assess the risk involved in moving planting stock north, even with data from long‐term provenance trials because mature trees may not be as susceptible to frost damage as seedlings and saplings. Generally, differences between provenances in the onset of cold hardiness in fall are greater than in the release of cold hardiness in spring, so a movement in latitude might have a bigger effect in changing susceptibility to early frosts in fall (Aitken & Hannerz, 2001). Cold hardiness heavily relies on the phenology of the onset and release of dormancy, and a trade‐off between growth and cold hardiness is usually driven by how long trees extend their growing season in the fall (Howe et al., 2003). The effect of climate change in fall usually gets less attention than other seasons even though fall events can have an important ecological impact (Gallinat, Primack, & Wagner, 2015).

While growth performance of white spruce provenances has been well studied, there is a lack of understanding of which physiological and anatomic traits are responsible for those genetic population differences. Trade‐offs between growth and cold hardiness or drought resistance could pose an additional challenge for forest managers to maintain the productivity and health of our forests under climate change. The research approach of this study was to screen groups of contrasting provenances from a wide variety of climatic source environments for a broad suite of physiological and anatomical traits that are putatively adaptive. To further enhance the probability of finding trade‐offs and discover genetic differentiation in adaptive traits, we selected provenances with contrasting combinations of growth and survival in a common garden field trial. The specific goals were to (1) quantify genetic population differentiation among provenances from across the entire range of the species for a wide selection of hydraulic, anatomical, and cold hardiness traits, (2) detect possible relationships among resistance to climate (cold and drought), tree growth, and survival, and (3) analyze how these traits are related to the climate of origin of the provenances. The results of this range‐wide experiment could point to key traits for climate adaptation that could serve as a reference for more geographically limited studies with higher sample densities to support regional assisted migration prescriptions.

## 2 METHODS

### 2.1 Plant material

Plant material for this study came from two contiguous white spruce (*Picea glauca* [Moench] Voss) trials in a single site in central Alberta, Canada (55°17′N, 113°10′W). Both trials belong to a provenance trial experiment described in detail by Rweyongeza et al. (2007). One of the trials is planted with provenances from only the province of Alberta, while the other trial has provenances covering the whole Canadian distribution of white spruce, with some provenances present in both trials. Both trials have five blocks each, with nine trees per block for the Alberta trial and five trees per block for the Canada‐wide trial. They were established in 1982 with four‐year‐old seedlings. Height was assessed after 32 growing seasons in the field, and survival was calculated as the ratio of living trees to the total planted.

**Figure 1:**
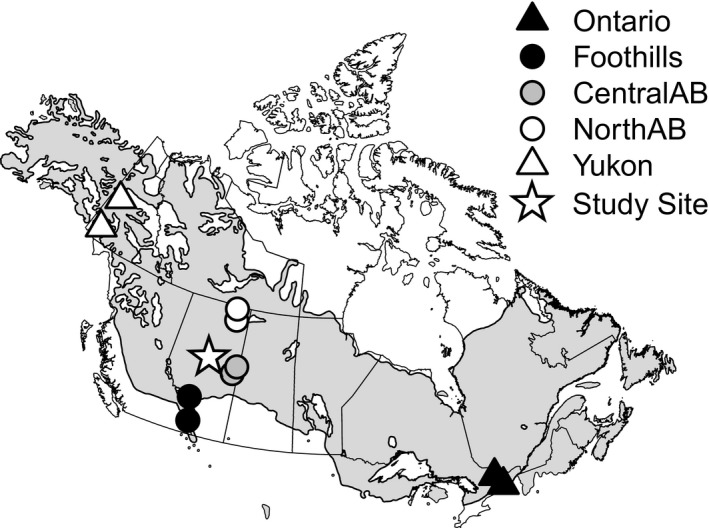
Locations of origin of provenances and the common garden test site where they were grown. The area in gray delineates the species range of white spruce

We selected two provenances from five distinct regions that covered most of the white spruce distribution (Figure 1, Table 1). The provenance selections for Alberta corresponded to the three regions that showed genetic differences in previous studies (Rweyongeza et al., 2007): northern Alberta (nAB), central Alberta (cAB), and Foothills (FH). The other two provenances belong to climatically and geographically different parts of the species range: Yukon (YU) in the north and Ontario (ON) in the south. The same sample trees were used for all measurements so that an individual comparison between variables was possible. A total of seven trees (at least one from each block) were selected for each provenance, except for provenances nAB.2 and cAB.2 that were present in both trials, and for which five trees from each trial and provenance were selected. The sample size was reduced for time‐consuming anatomic measurements (for details, see notes in Table 2). Climate data for the provenances and study site were extracted with the software ClimateNA v5.21, available at http://tinyurl.com/ClimateNA (Wang, Hamann, Spittlehouse, & Carroll, 2016). We used the standard reference normal period of 1961–1990 as a representation of the climate of origin to which populations are putatively adapted. The period has good weather station coverage and largely precedes a strong anthropogenic warming signal.

**Table 1:** Geographic location of the provenance origins and the common garden test site used in this study

Location	Latitude	Longitude	Elevation (m)
nAB.1	58.73	−111.25	235
nAB.2	59.88	−111.72	813
cAB.1	54.37	−110.75	396
cAB.2	54.63	−110.22	610
FH.1	51.40	−115.22	1750
FH.2	49.65	−114.62	1585
ON.1	45.97	−77.43	170
ON.2	45.50	−77.02	121
YU.1	61.35	−139.00	792
YU.2	64.02	−139.00	609
Site	55.27	−113.16	635

**Table 2:** Least squares means of field performance, anatomy, hydraulic, and cold hardiness traits. Individual provenances that have the same letter (in columns) are not significantly different at *p* < .05. Absence of letters for a trait indicates that there were no significant differences among provenances after an experiment‐wise α‐level adjustment for 45 pairwise comparisons

Provenance	Height	Survival	Cold30	Cold50	*A*_L_:*A*_X_	*K*_L_	*K*_S_	P50	Emb_Nat_	Density	Tr_Diam_	Tr_Length_
nAB.1	7.9 a	97 ab	0	19 ab	37.4 ab	0.0136	0.478	−4.09	3.8	0.569	10.3	1.44
nAB.2	7.9 abc	97 a	0	19 ab	32.9 ab	0.0157	0.449	−4.25	7.4	0.581	11.4	1.29
cAB.1	10.9 d	98 ab	4	24 ab	37.0 ab	0.0120	0.420	−4.04	6.8	0.577	11.0	1.38
cAB.2	9.9 d	93 abc	2	49 ac	35.3 ab	0.0174	0.520	−4.28	1.9	0.544	10.7	1.46
FH.1	7.2 a	89 abc	2	44 ac	29.1 ab	0.0188	0.490	−4.34	12.1	0.552	10.5	1.24
FH.2	7.6 a	75 c	3	96 d	21.2 a	0.0180	0.336	−4.31	6.9	0.538	11.2	1.28
ON.1	10.3 d	76 bc	11	82 cd	45.9 b	0.0108	0.534	−3.91	26.3	0.551	11.0	1.38
ON.2	9.6 cd	84 abc	14	91 d	35.5 ab	0.0154	0.520	−4.14	3.3	0.547	10.3	1.46
YU.1	5.0 b	96 abc	0	16 ab	33.9 ab	0.0180	0.537	−4.20	4.2	0.555	10.4	1.30
YU.2	4.4 b	92 abc	0	1 b	30.5 ab	0.0186	0.461	−4.42	16.6	0.556	12.3	1.55

Tree height (m) and survival (%, *N* = 70 for nAB.2 and cAB.2; *N* = 45 for nAB.1, cAB.1, FH.1 and FH.2; *N* = 25 for ON.1, ON.2, YU.1 and YU.2); Tr_Length_ = Tracheid length (mm, *N* = 1 tree/provenance; 200 tracheids/tree); Tr_Diam_ = Tracheid diameter (μm, *N* = 2); Density = wood density (g/cm^3^, *N* = 7); *A*_L_:*A*_X_ = leaf‐to‐xylem area ratio (cm^2^/mm^2^, *N* = 4); Emb_Nat_ = native embolism (%, *N* = 7); *K*_S_ = xylem‐specific maximum conductivity (mg mm^−1^ s^−1^ kPa^−1^, *N* = 7); *K*_L_ = leaf‐specific maximum conductivity (mg mm^−1^ s^−1^ kPa^−1^, *N* = 4); P50 = pressure at which 50% of the conductivity is lost (MPa, *N* = 7); Cold30 and Cold50 = frost damage at −30 and −50°C, respectively (%, *N* = 7).

### 2.2 Hydraulic measurements

Samples for hydraulic measurements were collected between 19 May and 8 June 2015. Distal branches from sun‐exposed parts of the tree crown were cut with a pole pruner. The branches were packed in plastic bags with wet paper towels to avoid desiccation and transported to a cold room (+4°C) the same day. All hydraulic measurements were made within 1 week after collection. We follow methodology described in Hacke and Jansen (2009) and Schoonmaker, Hacke, Landhausser, Lieffers, and Tyree (2010) for conductivity measurements. Briefly, we cut approximately 15‐cm‐long branch segments under water and attached them to a conductivity apparatus that applied a 20 mmol/L KCl+ 1 mmol/L CaCl_2_ solution under controlled pressure to the segment. The outflow was measured every 10 s with an electronic balance (CP225D; Sartorius, Göttingen, Germany) until it stabilized. The average of the last five measurements was used to calculate hydraulic conductivity (*K*_H_) with the following expression:

$$K_{H}=\frac{\text{Water flow}\times \text{segment length}}{\text{Pressure head}}$$

Hydraulic conductivity was first measured to assess the native conductivity of the segments in the field. Then, samples were subjected to a partial vacuum in the measuring solution to remove any native embolism and determine the maximum conductivity. Native embolism was calculated as the ratio of the initial to the maximum conductivity. Vulnerability to cavitation was assessed by applying a known pressure to the segment using a centrifuge and calculating the percentage loss of conductivity (PLC) relative to the maximum conductivity. This procedure was repeated for six pressure levels from −2 to −7 MPa, and the results were fitted to two commonly used functions: the Weibull function (Cai, Li, Zhang, Zhang, & Tyree, 2014) and the exponential sigmoidal function (Pammenter & Vander Willigen, 1998). The best fitted function was selected in each individual case. From these curves, we calculated the pressure at which the xylem loses 50% of its maximum conductivity (P50). Maximum *K*_H_ was used to calculate xylem‐area‐specific conductivity (*K*_S_) and needle‐area‐specific conductivity (*K*_L_). Xylem area (*A*_X_) was measured in the center of the segments using a stereomicroscope (MS5, Leica, Wetzlar, Germany). For the estimation of needle area (*A*_L_), we first measured the projected area of a subset of needles, and then, the same subset was weighted after drying the needles in an oven. Using the ratio of area to weight from the subset of needles, we could estimate the total area of the needles distal to the segment by drying and weighting the remaining needles. *K*_S_ and *K*_L_ were then calculated according to Tyree and Zimmermann (2002):

$$K_{S}=\frac{K_{H}}{A_{X}}$$

$$K_{L}=\frac{K_{H}}{A_{L}}$$

Lastly, the ratio of leaf area to xylem area (*A*_L_:*A*_X_) was also calculated as a measure of hydraulic efficiency.

### 2.3 Wood anatomy

The same segments used for hydraulic measurements were also used for wood anatomy analysis. Tracheid lumen diameter was measured for the most recent complete two rings of each segment using a radial file of three cells wide with images taken with a Leica DM3000 microscope at 20× magnification. Only noncompression wood was analyzed. The hydraulically weighted mean lumen diameter (*T*_diam_) was calculated using the Hagen–Poiseuille formula:

$$T_{\mathrm{diam}}={\left(\frac{\sum d^{4}}{n}\right)}^{1/4}$$

where *d* is tracheid diameter and *n* is the total number of tracheids measured. Tracheid length (*T*_length_) was measured using chemical macerations following Schoonmaker et al., 2010. Wood sections corresponding to the outer rings of each segment were digested in a 1:1 mixture of 80% glacial acetic acid and 30% hydrogen peroxide at 60°C for 48 hr. Macerated tissue was analyzed with a light microscope at 25× magnification to measure the length of 200 tracheids for each sample. All the image analyses were performed with ImagePro Premier (Media Cybernetics, Silver Spring, MD, USA) software.

### 2.4 Cold hardiness

Healthy, sun‐exposed branches were collected for cold hardiness measurements from the same trees on 22 September and brought to a cold room (+4°C) in plastic bags with wet paper towels within the same day. The whole‐plant freeze testing method (Burr et al., 2001) was used to estimate frost damage. Distal parts of the branches of approximately 20 cm length were frozen at different temperatures in a programmable freezer (85‐3.1A; ScienTemp, Adrian, MI, USA). We used a cooling rate of 5°C/hour and maintained the samples at the test temperature for one hour, followed by warming up to room temperature at the same rate of 5°C/hr. Three test temperatures were used: −30°C, −40°C, and −50°C. After the freezing treatment, samples were transferred to a growth chamber where they were stored in transparent plastic bags with wet paper towels. Frost damage in the needles was assessed visually 2 weeks after the treatment as a percentage of discolored needle surface.

### 2.5 Data analysis

All statistical analyses were conducted with the R v3.2 programming environment (R Core Team 2017). We used a mixed‐effects model to calculate the means of the provenances using the variable of interest as a fixed effect and block as a random effect with the *lme4* package (Bates, Mächler, Bolker, & Walker, 2015). Means were calculated as least squares means with the *lsmeans* package (Lenth, 2016). Multiple comparisons of the means were carried out with the general linear hypothesis method of the *multcomp* package (Hothorn, Bretz, & Westfall, 2008). Differences among provenances in survival were tested for statistical significance with Fischer's exact *z*‐test and Holm's adjustment for multiple inference, implemented with the *p.adjust* function of the R base package. We visualized the effect of the climate of origin on the performance in the field using a multivariate indirect gradient analysis. The ordination space consisted of standardized values of height and survival, defined as standard deviations from the overall experimental mean set to zero. We calculated the individual correlations of each climatic variable with this ordination space using the function *vf()* from the package *ecodist* (Goslee & Urban, 2007) and plotted them as vectors in the figure. To analyze trade‐offs and relationships among specific hydraulic and anatomical traits, we conducted boundary analyses with quantile regressions using the function *rq()* from the package *quantreg* (Koenker, 2017). A quantile regression can be used when a response cannot change by more than some upper limit, but may change by less when other unmeasured factors are limiting (Cade & Noon, 2003). A quantile regression is similar to a linear regression, but it allows for the estimation of a quantile instead of the average. In this case, we calculated the 5% (P50) and 95% (wood density) quantiles. This regression can identify a region of a scatter plot where it is very rare to find points and can point to a trade‐off even if the overall correlation between variables for the majority of the sample points is weak.

## 3 RESULTS

**Figure 2:**
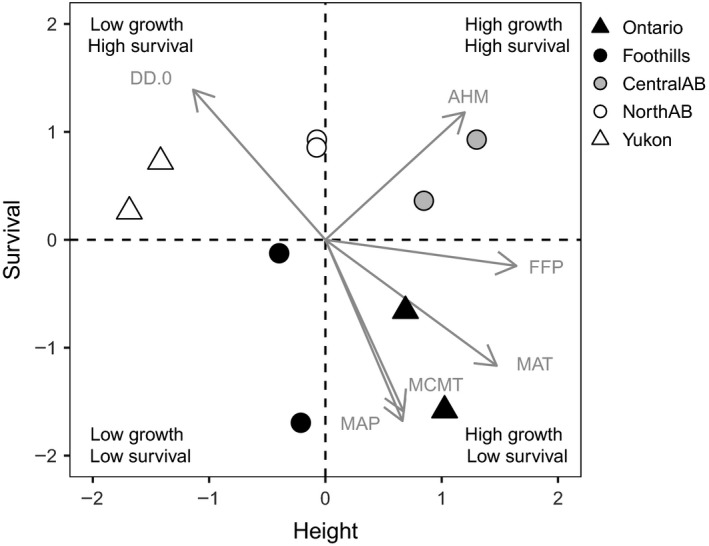
Performance of the provenances at the study site. Height and survival are expressed in units of standard deviations from an overall mean of zero. The vectors represent significant associations of the climate of the provenance origin with growth and survival in an indirect gradient analysis, where DD.0 = degree‐days below 0°C, AHM = annual heat–moisture index, FFP = frost‐free period, MAT = mean annual temperature, MCMT = mean coldest month temperature, and MAP = mean annual precipitation

Provenance field testing results conformed to the expectation that “local is best,” with provenances from the vicinity of the planning site showing the highest growth and good survival (Figure 2, upper‐right quadrant). Provenances from the Alberta Foothills represented the other extreme, with below‐average growth and survival. The remaining six provenances showed a trade‐off between growth and survival, with provenances from Ontario having high growth but relatively low survival and provenances from northern regions (Yukon and Northern Alberta) exhibiting high survival but lower growth. The observed differences in growth and survival among individual provenances were statistically significant as indicated in Table 2, and they provide a reference to interpret the importance of physiological and anatomical adaptive traits as causes of population performance in the field.

**Figure 3:**
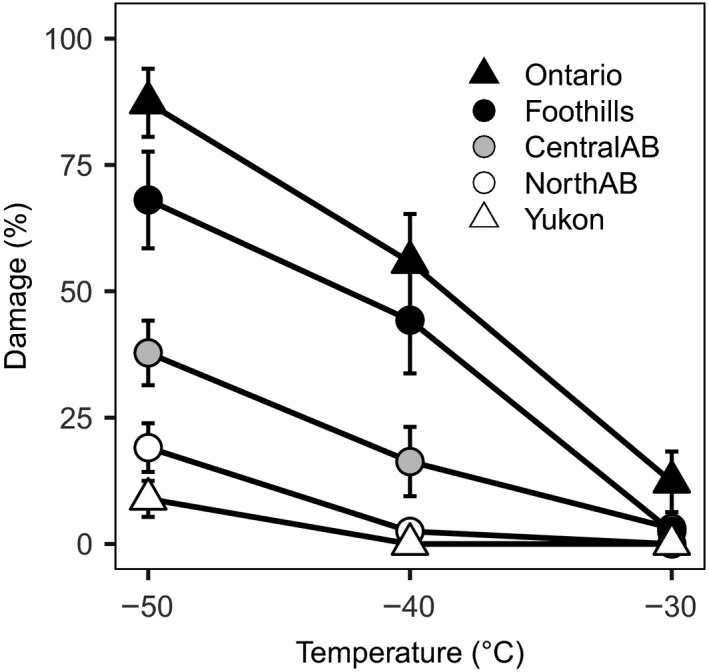
Percentage of damaged tissue shown by the different regions at three experimental freezing temperatures. Error bars represent standard errors of the mean. Samples were collected on 22 September

Among the physiological and wood anatomical variables measured, we found the strongest differentiation in cold hardiness (Figure 3, Table 2). At the time of sampling, the −50°C test temperature resulted in the greatest range of observed damage from 1% to 96% in one of the Yukon (YU.2) and one of the Foothills (FH.1) provenance samples, respectively. Provenances within the same region generally behaved similarly, except for the Alberta Foothills, which showed a statistically significant difference between its provenances. Most of the provenances showed good resistance to −30°C temperatures at the time of assessment in mid‐September, and only Ontario provenances showed significant damage (12%). We observed no significant differences among provenances in hydraulic and anatomic variables putatively related to adaptation to drought, except for leaf‐to‐xylem area ratio, due to high intrapopulation variance (Table 2).

**Figure 4:**
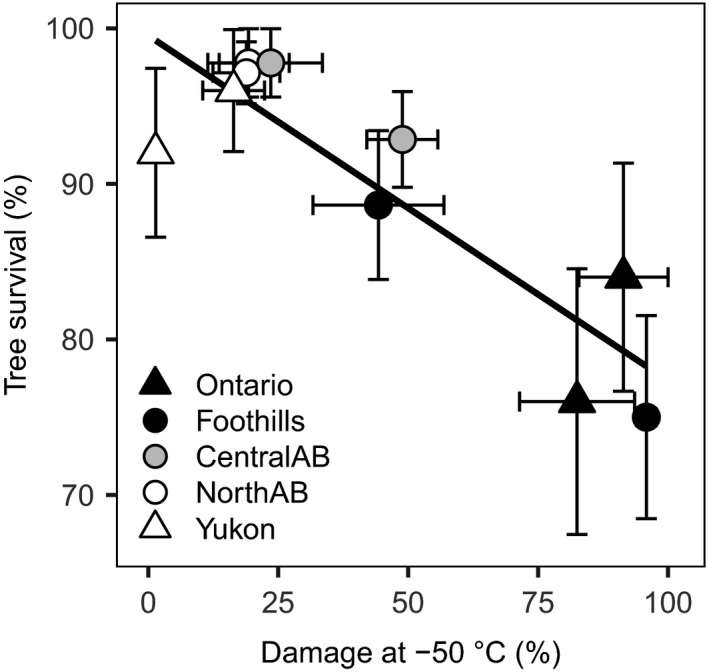
Tree survival was correlated to cold damage at a test temperature of −50°C (*R* ^2^ = .746, *p* < .001). Error bars correspond to one standard error

**Figure 5:**
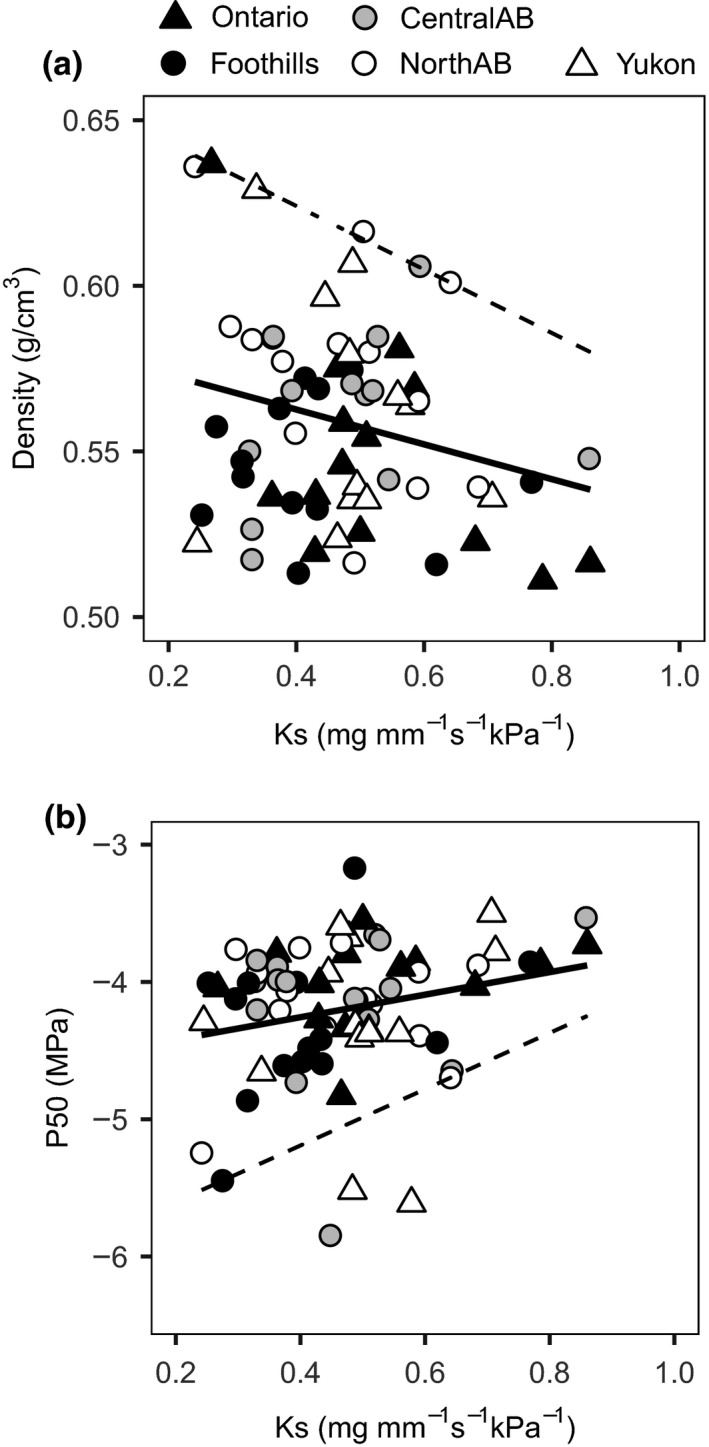
Relationship of individual values of xylem‐specific conductivity (*K*_S_) and (a) wood density (*R* ^2^ = .077, *p* = .013) and (b) vulnerability to cavitation expressed as P50, the xylem pressure inducing 50% loss of hydraulic conductivity (*R* ^2^ = .048, *p* = .042). The dashed lines represent the 95% (a) and 5% (b) quantile regression lines to illustrate the scarcity/absence of data points in the upper‐right (a) and lower‐right (b) corners

Frost hardiness was strongly related to survival (Figure 4), but it did not show a significant trade‐off with tree height (Table 3). There was a significant correlation between height and both *K*_L_ and P50, but we did not find any relationship between survival and hydraulic variables (Table 3). Despite weak correlations, the boundary analysis suggested trade‐offs between xylem‐specific conductivity (*K*_S_) and wood density (Figure 5a) and between xylem‐specific conductivity and the pressure at which the xylem loses 50% of its maximum conductivity (*P50*) (Figure 5b). Using quantile regressions, we are able to estimate a boundary (dashed line in Figure 5) beyond which values are very rare to find. Low transport efficiency (low *K*_S_ values) was associated with a wide range of both *P50*s and xylem densities, but the most hydraulically efficient branches were always characterized by vulnerable xylem and low densities, indicating that an ideal combination of high efficiency and high cavitation resistance is not possible (note the absence of data points in the top right in Figure 5a and bottom right in Figure 5b). The leaf‐to‐xylem area ratio appeared as an influential trait for hydraulic variables as it was significantly correlated to both efficiency (*K*_S_ and *K*_L_) and cavitation resistance (*P50*; Table 3). Leaf‐specific conductivity was strongly correlated to *P50*, so that the more vulnerable branches had also a poorer ability to provide water to the needles. Wood density was the most influential among anatomic variables, as it was significantly correlated to tree survival and cold hardiness. Tracheid size was not related to any variable measured.

**Table 3:** Pearson correlation coefficients for the mean values of provenances for anatomy, hydraulic, cold hardiness, and performance variables. Statistically significant correlations at *p* < .05 are highlighted in bold

	Height	Survival	Cold30	Cold50	*A*_L_:*A*_X_	*K*_L_	*K*_S_	P50	Density	Tr_Diam_
Survival	−0.19									
Cold30	0.61	−0.61								
Cold50	0.49	−**0.88**	**0.75**							
*A*_L_:*A*_X_	0.50	0.12	0.44	−0.05						
*K*_L_	−**0.71**	0.05	−0.49	−0.15	−**0.79**					
*K*_S_	0.03	0.17	0.29	−0.10	**0.68**	−0.12				
P50	**0.67**	−0.12	0.56	0.26	**0.82**	−**0.94**	0.30			
Density	0.07	**0.72**	−0.34	−**0.67**	0.30	−0.43	−0.08	0.26		
Tr_Diam_	−0.39	−0.06	−0.31	−0.30	−0.29	0.20	−0.44	−0.44	0.13	
Tr_Length_	0.03	0.15	0.17	−0.18	0.33	−0.15	0.24	0.02	−0.05	0.30

Cold30 and Cold50 = frost damage at −30 and −50°C, respectively, A_L_:A_X_ = leaf area to xylem area, *K*_L_ = leaf‐specific conductivity, *K*_S_ = xylem‐specific conductivity, P50 =  vulnerability to cavitation expressed as the pressure at which 50% of the maximum conductivity is lost, Density = wood density, Tr_Diam_ = tracheid diameter, Tr_Length_ = tracheid length.

**Figure 6:**
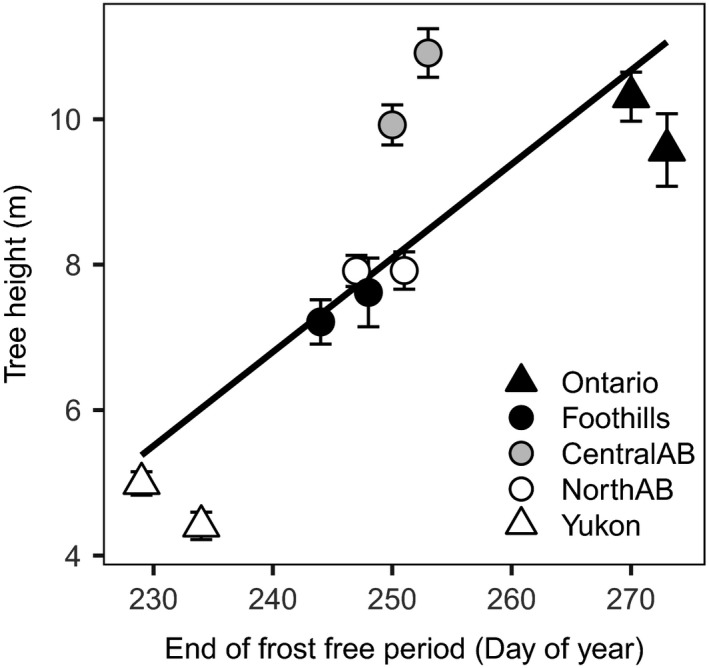
Relationship between tree height and the average date of the first frost event at the source of the provenances (*R* ^2^ = .616, *p* = .004), showing the effect of the end of the growing season on tree growth. Error bars represent the standard error of the mean

The climate of origin was strongly associated with the field performance of provenances. Climate vectors significantly correlated to height, survival, or a combination of both traits as per indirect gradient analysis. This is displayed in Figure 2, where the vector length with respect to each axis represents the strength of the association. The variables that were most strongly correlated with survival are, in this order, mean average precipitation, mean coldest month temperature, and degree‐days below 0°C, suggesting that provenances with better survival came from dry climates with cold and long winters. For growth, the most important predictor variables were frost‐free period and mean annual temperature at the origin of the seed source. If we evaluate the influence of the beginning and end of the frost‐free period separately, we can see the higher influence of the end of growing season variable, which is also statistically significant (Table 4, Figure 6). Annual heat–moisture index was not significantly correlated to either height or survival alone (Table 4), but it was associated with high combined survival and growth of central Alberta provenances (Figure 2).

**Table 4:** Pearson correlation coefficients for the relationship between climate variables and anatomy, hydraulic, cold hardiness, and performance variables. Statistically significant correlations at *p* < .05 are highlighted in bold

	Height	Survival	Cold30	Cold50	*A*_L_:*A*_X_	*K*_L_	*K*_S_	P50	Density	*T*_Diam_	*T*_Length_
Latitude	−0.68	−0.76	−**0.85**	−**0.92**	−0.17	0.35	−0.03	−0.44	0.49	0.42	0.19
MAT	0.78	−0.67	**0.91**	**0.87**	0.31	−0.47	0.09	0.54	−0.41	−0.41	−0.02
MWMT	0.69	−0.20	0.77	0.37	0.70	−0.74	0.30	0.68	0.11	−0.13	0.51
MCMT	0.46	−**0.81**	0.69	**0.90**	−0.13	−0.07	−0.13	0.19	−0.65	−0.34	−0.33
TD	−0.14	0.70	−0.33	−0.71	0.43	−0.25	0.26	0.11	0.68	0.27	0.54
MAP	0.47	−**0.85**	**0.84**	**0.88**	0.12	−0.23	0.07	0.30	−0.56	−0.24	−0.11
MSP	0.58	−0.65	0.75	0.74	0.22	−0.24	0.21	0.31	−0.49	−0.36	−0.15
AHM	0.54	0.53	−0.06	−0.20	0.33	−0.44	0.01	0.45	0.43	−0.38	0.05
SHM	−0.24	0.61	−0.36	−0.62	0.21	−0.18	0.01	0.05	0.66	0.32	0.42
DD < 0	−0.65	0.74	−0.76	−**0.91**	−0.04	0.24	0.06	−0.35	0.55	0.43	0.26
DD > 5	0.76	−0.34	**0.88**	0.53	0.67	−0.71	0.31	0.70	−0.04	−0.25	0.40
bFFP	−0.74	0.01	−0.61	−0.22	−0.67	0.73	−0.22	−0.61	−0.22	0.07	−0.59
eFFP	**0.81**	−0.49	**0.89**	0.71	0.51	−0.67	0.16	0.66	−0.13	−0.35	0.17
FFP	**0.82**	−0.27	**0.80**	0.50	0.62	−0.74	0.20	0.67	0.05	−0.23	0.40

Cold30 and Cold50 =  frost damage at −30 and −50°C, respectively, *A*_L_:*A*_X_ = leaf area to xylem area, *K*_L_ = leaf‐specific conductivity, *K*_S_ = xylem‐specific conductivity, P50 = vulnerability to cavitation expressed as the pressure at which 50% of the maximum conductivity is lost, Density = wood density, *T*_Diam_ = tracheid diameter, *T*_Length_ = tracheid length. MAT= mean annual temperature, MWMT = mean warmest month temperature, MCMT = mean coldest month temperature, TD = continentality or temperature difference between MWMT and MCMT, MAP = mean annual precipitation, MSP = May‐to‐September precipitation, AHM = annual heat–moisture index (MAT + 10)/(MAP/1000), SHM = summer heat–moisture index MWMT/(MSP/1000), DD<0 = degree‐days below 0°C, DD>5 = degree‐days above 5°C, bFFP = the day of the year on which FFP begins, eFFP = the day of the year on which FFP ends, FFP = frost‐free period.

**Figure 7:**
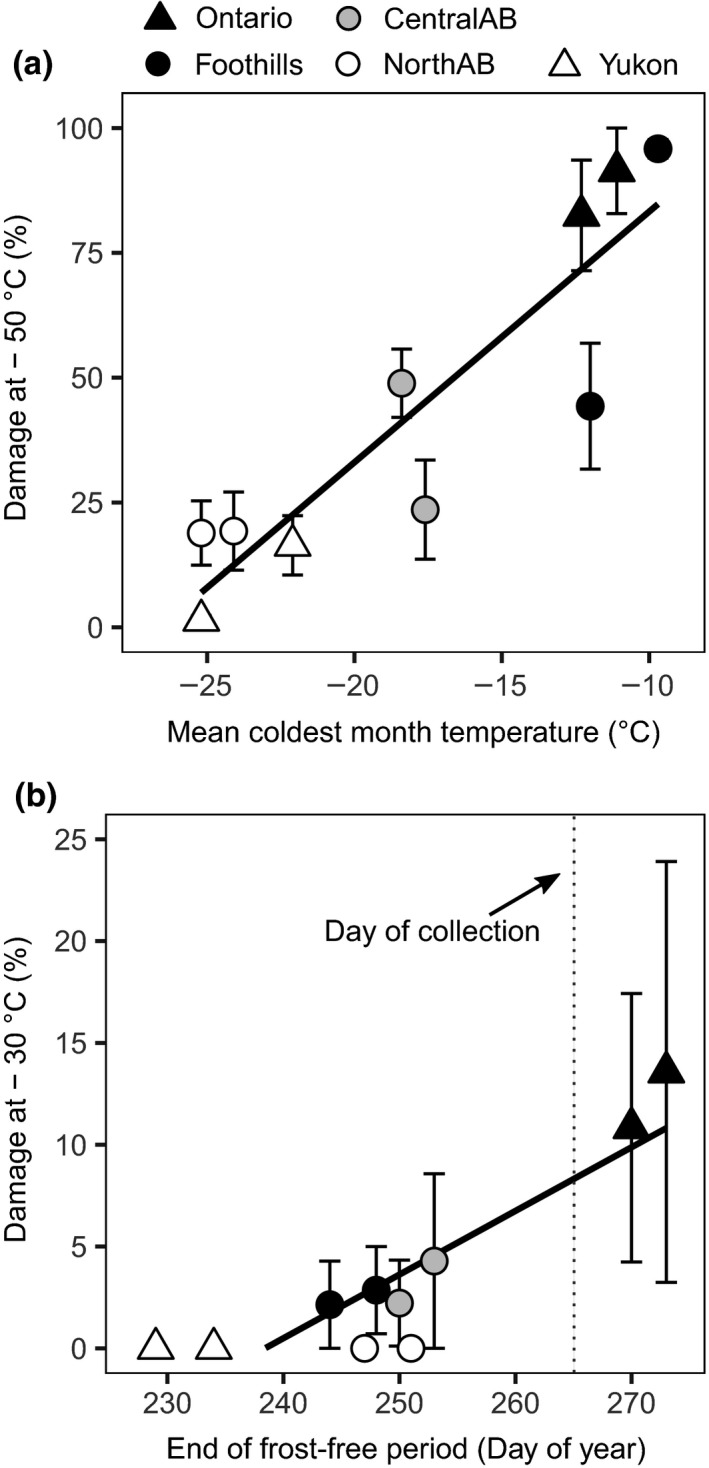
Relationship between cold hardiness (on the *y*‐axis) and source climate (on the *x*‐axis). (a) Cold damage at −50°C showed a high correlation to the mean coldest month temperature (*R* ^2^ = .784, *p* < .001). (b) Cold damage at −30°C as a function of the average day of the year when the first frost event occurs. Samples were collected on day 265 (22 September, dashed line). Only Ontario provenances (closed triangles) showed damage significantly greater than 0, consistent with the absence of frost at their native environment at that time of year. Error bars correspond to one standard error

Cold hardiness was also associated with the climate of origin. Damage at −50°C was mostly related to day length and winter temperatures, having the highest correlations with latitude, mean coldest month temperature, and degree‐days below 0°C (Figure 7a, Table 4). Damage at −30°C was more correlated to variables corresponding to annual temperatures like evapotranspiration and mean average temperature and also to the date of the first frost event (Figure 7b, Table 4). It is important to note that by the time of sample collection (day of year 265), the first frost event at the location of origin for an average year would have already happened for all provenances except for those originating from Ontario (Figure 7b). We did not find any significant associations between climate at the origin of seed sources and hydraulic or anatomic variables (Table 4).

## 4 DISCUSSION

### 4.1 Local adaptation and trade‐offs

Although this study was not designed to study local optimality or to be used to derive practical assisted migration guidelines, provenances that originated near the vicinity of the study site showed the best combination of long‐term performance in growth and survival in the field. Local provenances (central Alberta) originated slightly southeast of the study site which corresponds to a short northwest transfer as was recommended by Gray et al., 2016 to account for climate change that has already occurred over the last several decades. The provenances that showed both poor growth and low survival came from the Alberta Foothills (lower‐left quadrant in Figure 2). This area corresponds to relatively mild climate conditions both in summer and winter due to the presence of adiabatic heating on the leeward side of the Rocky Mountains and the summer jet stream of ocean origin bringing precipitation. Possible introgression with Engelmann spruce, native to higher elevation than white spruce, would enhance its adaptation to these mountainous conditions at the cost of lower growth (De La Torre, Wang, Jaquish, & Aitken, 2014). Liepe, Hamann, Smets, Fitzpatrick, and Aitken (2016) also reported that high‐elevation sources of spruce had short growing seasons, lacked strong cold tolerance, and tended to be poor growers. The other six provenances in our study showed a clear trade‐off between growth and survival. Northern provenances (Yukon and Northern Alberta), with the coldest winters and lowest precipitation, exhibited high survival but inferior growth. For Ontario provenances, coming from areas with more favorable conditions for plant growth where inter‐ and intraspecies competition is presumably more important, the opposite was observed: an emphasis of growth over safety.

### 4.2 Strong population differentiation in cold hardiness

The adaptive trait with the strongest genetic population differentiation was cold hardiness, and trait values showed strong associations to latitude and the mean coldest month temperature of seed origin. Cold hardiness was measured in branches collected around mid‐September, and at this time of year, only provenances from Ontario exhibited some frost damage after exposure to –30°C. Ontario provenances usually do not experience any frost at this time of year at their location of origin. Hence, there is no need to develop cold hardiness so early in the fall when trees grow in their native climate. The other provenances do regularly experience mild frost events earlier in September in their native climate and exhibited no significant damage when exposed to –30°C at the test site. The fact that cold hardiness patterns could largely be explained by the climate of the provenance origins indicates that cold hardiness was genetically differentiated among populations, presumably mediated by day length, and that there is little potential for phenotypic plasticity in response to climate conditions. These results conform to many other studies (Howe et al., 2003 and literature cited therein). Of all the traits measured in this study, cold hardiness appeared as the most important adaptive trait, clearly linked to survival of provenances in the field. While cold hardiness was not significantly correlated to tree height, we found a strong correlation of height with the end of the growing season at the provenance origin. This suggests that the timing of the onset of dormancy plays an important role in the growth potential of white spruce.

### 4.3 Wood anatomical and hydraulic parameters

While it seems that patterns in survival and tree height could largely be explained by cold tolerance and growing season length, respectively, we also measured a wide range of hydraulic and wood anatomical parameters, putatively related to drought resistance. Wood density emerged as a potentially influential variable as it was correlated with survival and cold resistance. Lighter wood may increase the risk of stem breakage (Spatz & Bruechert, 2000), which may be important under heavy snow loads (Hlasny, Kristek, Holusa, Trombik, & Urbancova, 2011). High wood density also provides a stronger defense against pathogens as well as lower vulnerability to drought stress (Chave et al., 2009; Hacke, Sperry, Pockman, Davis, & McCulloch, 2001; Hacke et al., 2015; Rosner et al., 2014). Higher wood densities are often found in environments that are associated with stress. Positive correlations of wood density with survival and cold hardiness seem consistent with these reports.

Tree height was negatively correlated with leaf‐specific conductivity (*K*_L_), that is, taller trees tended to show lower *K*_L_ values than shorter trees. Lower values of *K*_L_ corresponded to higher needle area distal to the section measured relative to its ability to transport water. *K*_L_ was mostly regulated by needle area rather than by a change in hydraulic conductivity as we can see from the significant correlation between *K*_L_ and *A*_L_:*A*_X_ and the absence of a correlation between *K*_L_ and *K*_S_ (Table 3). Similar findings were reported from studies on pine populations sampled across climate gradients (Lopez, Cano, Choat, Cochard, & Gil, 2016; Martínez‐Vilalta et al., 2009). At a particular transpiration rate (*E*), a branch with higher *K*_L_ will be able to maintain a smaller water potential gradient (ΔΨ) than a branch with lower *K*_L_, because *E *= *K*_L_ ΔΨ (Tyree & Zimmermann, 2002). While having high *K*_L_ may seem conservative and advantageous from a hydraulic perspective, it comes with the disadvantage of having less photosynthetic area or having to invest more resources into nonphotosynthesizing xylem tissue to increase transport capacity.

In our study, Yukon and Foothill provenances exhibited the highest *K*_L_ values. Both Yukon and Foothills have the shortest frost‐free periods. Trees in these regions must deal with frozen soils for the longest time, and they may also experience many freeze–thaw events in spring. Drought stress can develop in the winter when water uptake from a frozen/cold soil is impaired while needles continue to lose water (Mayr, Hacke, Schmid, Schwienbacher, & Gruber, 2006). Having a higher *K*_L_ may reduce the tension in the xylem during this period and may therefore be advantageous over a greater emphasis on leaf area and growth potential.

Hydraulic conductivity standardized by xylem area (*K*_S_) was not correlated with growth or survival, but showed complex relationships with wood density and P50. The boundary analysis approach shown in Figure 5 illustrates that high wood densities and *K*_S_ values were mutually exclusive. This points to a strength versus hydraulic efficiency trade‐off and is understandable given constraints arising from the geometry of tracheid‐based xylem (see detailed discussion in Pittermann, Sperry, Hacke, Wheeler, & Sikkema, 2006; Hacke et al., 2015). High hydraulic conductivity (*K*_S_) values also came at the expense of increased vulnerability to cavitation. The safety versus efficiency trade‐off is complex (Gleason et al., 2016), but our boundary analysis indicates that certain trait combinations are unattainable despite high within‐population variation in either of these traits.

## 5 CONCLUSION

In this range‐wide common garden study, we identified traits that could play an important role in trade‐offs between growth potential and resistance to climatic stress. We should note that the growing conditions of the test site can affect what trade‐offs are revealed. Differences in adaptive traits that did not have an effect on growth and survival could be consequential under different growing environments. Wood density and leaf‐specific conductivity were identified as potentially important traits, but fall cold hardiness stood out as a key trait for tree survival and also showed easily interpretable associations with climate of the provenance origin. Furthermore, the relation between height and the end of the growing season points to the importance of the timing of growth cessation in the growth potential of white spruce. Therefore, regional studies that make use of a higher sampling density to develop assisted migration guidelines should focus on measuring the effects of seed transfers on fall phenology and match provenances to new locations so that their synchronization of the onset of frost hardiness matches new environmental conditions under climate change.

## CONFLICT OF INTEREST

None declared.

## AUTHORS’ CONTRIBUTIONS

AH, UH, and JS conceived the study and designed the methodology. JS collected the data and led the writing of the manuscript. All authors analyzed the data and contributed to draft versions of the manuscript.
